# Preventing graft restenosis after coronary artery bypass grafting with tissue-type plasminogen activator

**DOI:** 10.1186/s40001-017-0259-8

**Published:** 2017-06-12

**Authors:** Ruixiong Li, Bin Lan, Tianxiang Zhu, Yanlong Yang, Muyan Cai, Zhongmin Fang, Chensheng Ma, Shu Chen

**Affiliations:** Cardiothoracic Surgery, Shantou Central Hospital and Affiliated Shantou Hospital of Sun Yat-sen University, Shantou, 515031 People’s Republic of China

**Keywords:** Coronary artery bypass grafting, Restenosis, Tissue-type plasminogen activator

## Abstract

**Objective:**

To explore the feasibility and safety of using tissue-type plasminogen activator (t-PA) to prevent graft restenosis after coronary artery bypass grafting (CABG).

**Methods:**

In this prospective observational study, 37 patients underwent CABG between June 2009 and May 2013. These patients were grouped according to the anti-coagulation strategy after surgery: t-PA (*n* = 12) and conventional treatments (*n* = 25). In the t-PA group, the patients received acetylsalicylic acid (ASA) and clopidogrel plus intravenous infusion of t-PA (0.25 mg/kg/day) starting at 24 h after surgery and that lasted for 3 days. In the conventional group, the patients received only ASA and clopidogrel. 64-row spiral computed tomographic coronary angiography was performed at 1 week, 1, and 3 months after surgery to evaluate the patency of the graft vessel.

**Results:**

The mean stenosis severity of the saphenous vein grafts was lower in the t-PA group compared with the conventional group at 3 months after surgery (*p* < 0.05), but there was no significant difference at 1 week and 1 month (*p* > 0.05). The patency rate of the grafts was not significantly different between the two groups at 1 week, 1, and 3 months after surgery (*p* > 0.05).

**Conclusion:**

Early application of t-PA after CABG was feasible and safe, and might help prevent early restenosis of SV grafts. Additional clinical randomized trials are necessary to address this issue.

## Background

Coronary artery bypass grafting (CABG) is widely used for the treatment of coronary heart disease (CHD) and remains the most common form of cardiac surgery [1]. Currently, more than 300,000 patients undergo CABG in the United States each year [2]. Although the short-term outcomes of CABG are generally excellent, patients remain at risk of future cardiac events due to progression of coronary disease and/or coronary bypass graft failure [3–5]. Postoperative occlusion of the grafts occurs in approximately 20% of vein grafts and about 5% when an internal mammary artery is used [6–8]. Aggressive risk factor reduction is recommended in patients with coronary heart disease to increase graft patency including aspirin, treatments for hypertension and serum lipids, no smoking, and serum glucose control [9].

Vein grafts are vulnerable to endothelial damage that may occur during the operation and by sudden exposure to the high-pressure and pulsatile arterial system [10, 11]. Platelet deposition occurs in areas of endothelial damage and initiates thrombus formation that begins during the operation [10].

Plasmin plays a key role in clot lysis and is expected to limit restenosis [10]. Active plasmin is generated from its inactive proenzyme, plasminogen, by an endogenous tissue plasminogen activator (t-PA) and a urokinase-type plasminogen activator (u-PA). Decreased expression of t-PA is associated with graft restenosis [12].

Elements of the fibrinolytic pathway are utilized as antithrombotic agents, but their systemic use at therapeutic doses can lead to uncontrolled bleeding or rebound thrombosis [13, 14]. Targeted delivery of uPA to arterial thrombi with coated catheters and t-PA delivery by a microporous catheter were developed as a bailout intervention for peripheral artery disease and to treat thrombus in coronary arteries [15]. Luminal exposure of pig vein grafts to t-PA enzyme was found to be sufficient to improve the thrombolytic activity of the grafts [16].

Nevertheless, there are only a few studies examining the use of t-PA after CABG. Therefore, the aim of the present study was to explore the feasibility and safety of using t-PA to prevent restenosis of the vein grafts after CABG.

## Patients and methods

### Patients

In this prospective observational study, 37 patients who underwent elective off-pump beating heart coronary artery bypass surgery at the Shantou Central Hospital and Affiliated Shantou Hospital of Sun Yat-sen University between June 2009 and May 2013 were included. Exclusion criteria were as follows: (1) history of heart surgery or emergency surgery; (2) pre-existing inflammatory diseases (infection, active arthritis, and malignancies); (3) use of anti-inflammatory drugs such as glucocorticoids; (4) respiratory insufficiency; (5) severe neurological diseases; (6) chronic liver diseases; (7) renal insufficiency; or (8) any heart congenital malformations.

This study was approved by the ethical committee of the Shantou Central Hospital and Affiliated Shantou Hospital of Sun Yat-sen University. Written informed consents were obtained from all patients prior to their enrollments.

### Grouping and treatments

The patients were grouped according to the treatment they received: the t-PA group (*n* = 12) and the conventional group (*n* = 25). The choice of therapy was made after discussion between the surgeon and patients about the potential benefits or bleeding tendency of preoperatively and postoperative anti-coagulation. The t-PA group received acetylsalicylic acid (ASA), clopidogrel, and intravenous infusion of t-PA (0.25 mg/kg/day) starting 24 h after operation and that lasted for 3 days. The conventional group only received ASA and clopidogrel.

### Surgery

Inhalation/intravenous general anesthesia was performed under normal temperature. The great saphenous vein (SV) was obtained. The left internal mammary artery (LIMA) was isolated by an incision in the middle of the sternum. Intravenous semi-heparinization (100–200 U/kg) was performed to maintain the activated coagulation time (ACT) at 250–300 s. The target vessel was fixed, and the distal end was sutured with a 7–0 prolene thread. The lateral wall of the ascending aorta was clamped, and then punctured. The proximal end was sutured with a 6–0 prolene thread. All the procedures were performed by the same experienced surgeon. All patients were transferred to the ICU after surgery. Data were recorded, including the operation time, number of grafts transplanted, volume of postoperative drainage, and amount of transfusion.

### Early postoperative anti-coagulation strategy

Oral or nasal intake of aspirin and clopidogrel along with calcium antagonists and lipid-lowering drugs were given to all patients starting at 24 h after surgery. After the patients in the t-PA group were confirmed to be without bleeding tendency (hemorrhage was less than 20 mL/H, and PT and APTT were normal) 24 h after surgery, intravenous infusion of t-PA (Actilyse™; Boehringer Ingelheim, Ltd., Ingelheim am Rhein, Germany) at 0.25 mg/kg/day diluted to 1 mg/mL in normal saline was administered for 3 days.

### Postoperative coronary angiography

In all patients, 64-row spiral CT coronary angiography (Light Speed VCT; GE Healthcare, Waukesha, WI, USA) was performed 1 week, 1, and 3 months after surgery. The patients were asked to fast for at least 4 h before the operation. An iodine allergy test was performed. Heart rate (HR) was controlled at <70 beats/min. No arrhythmia was found. A β-blocker was sublingually administered to patients with HR >70 beats/min.

Using the technique of dual high-pressure syringe, a total amount of 55–75 mL of the non-ionic contrast medium Ultravist 370 (Bayer HealthCare Pharmaceuticals, Montville, NJ, USA) and 30–40 mL of normal saline were infused at 3.5–4.5 mL/s via an upper extremity distal vein or the median cubital vein. Meanwhile, the ^Sure^Start™ technique was used to initiate the continuous dynamic scanning (a delay of 8 s was given to reduce radiation exposure). CT at 130–150 Hu of the ascending aorta was used as the cut-off to trigger scanning. The scanning parameters were as follows: tube speed of 0.4 s/rotation, slice of 0.5 mm, and pitch from 11.2 to 14.4. If patients had an HR >75 bpm or arrhythmia (1 premature beat or interval), the parameters were as follows: tube speed of 0.45 s/rotation, slice of 0.5 mm, and pitch of 11.2 to improve the resolution. The scanning range included regions from 10 to 15 mm over the left coronary artery to 10–15 mm below the apex (when observing the bridging vessels, the scanning started at 10–15 mm over the aortic arch).

The stenosis severity of the grafts was compared and calculated according to the following formula: stenosis severity = (the proximal normal vessel diameter of stenosis − the diameter of stenosis)/the proximal vessel diameter of the stenosis × 100%. Stenosis >50% was considered as significant stenosis, while stenosis <50% was considered as graft patency [17–20].

### Follow-up and observation indexes

Coronary CTA was performed during follow-up (1 week, 1, and 3 months after surgery) for patients from the both groups to observe their conditions of restenosis in the vessel bridges, hemorrhage, and embolism.

### Statistical analysis

Continuous data were presented as means and standard deviations (SD) and were compared with independent samples *t* test. Categorical data were presented as frequencies and were compared with the Chi-square test. All statistical analyses were performed using SPSS 18.0 (SPSS Inc., Chicago, IL, USA). Two-sided *p* values <0.05 were considered statistically significant.

## Results

### Characteristics of the patients

Thirty-seven patients (22 males and 15 females) aged 53–69 years, weighing 52–78 kg, and measuring 156–178 cm were included. Twelve patients (8 males and 4 females) aged 61.8 ± 7.9 years were included in the t-PA group, and 25 patients (14 males and 11 females) aged 61.2 ± 8.3 years were in the conventional group. There were no significant differences between two groups for gender, age, body weight, triglycerides, cholesterol, platelets, and days of aspirin discontinuation before the operation (all *p* > 0.05) (Table 1).

**Table 1:** Baseline data of the patients in the two groups

Parameters	t-PA group	Conventional group	*p*
Age (years)	61.8 ± 7.9	61.2 ± 8.3	0.84
Height (cm)	165.2 ± 6.2	167.5 ± 7.9	0.38
Weight (kg)	63.8 ± 10.6	68.4 ± 11.2	0.24
Triglycerides (mmol/L)	2.41 ± 0.61	2.62 ± 0.83	0.78
Cholesterol (mmol/L)	4.45 ± 1.51	4.31 ± 1.60	0.8
Blood platelets (×10^9^/L)	212.5 ± 75.8	191.1 ± 91.3	0.49
Days of aspirin discontinuation before surgery	6.5 ± 3.7	6.7 ± 3.8	0.88

### Perioperative measurements

A total of 101 grafts were transplanted for 37 patients. One graft, two grafts, and three grafts were performed on 1 case, 8 cases, and 28 cases, respectively. Among these grafts, 11 grafts from 34 LIMA and 23 from 67 SV were transplanted for the t-PA group patients compared to 23 grafts from LIMA and 44 from SV that were transplanted in the conventional group. There were no significant differences between the two groups for the operation time, mean number of grafts transplanted, volume of postoperative drainage, and volume of postoperative blood transfusion (*p* > 0.05) (Table 2).

**Table 2:** Comparisons of the perioperative measurements between the two groups

Parameters	t-PA group	Conventional group	*p*
*N*	12	25	
Operation time (min)	232.5 ± 37.2	245.1 ± 42.1	0.38
Grafts transplanted	2.8 ± 0.7	2.7 ± 0.6	0.65
Volume of postoperative drainage (mL)	515.4 ± 135.5	539.8 ± 154.2	0.64
Volume of postoperative blood transfusion (mL)	1292.6 ± 463.7	1235.1 ± 486.4	0.73

### Postoperative measurements

The patency rate of the grafts (including LIMA and SV) at 1 week, 1, and 3 months after surgery was not significantly different between the two groups (*p* > 0.05) (Table 3), but the mean stenosis degree at 3 months after surgery was significantly lower in the t-PA group compared with the conventional group (*p* < 0.05), while the stenosis degree 1 week and 1 month after surgery was not significantly different (*p* > 0.05). Postoperative blood pressure, triglycerides, cholesterol, and platelets were not significantly different between the two groups (*p* > 0.05).

**Table 3:** Comparison of the patency rate and stenosis degree of the grafts between the two groups

Parameters		t-PA group	Conventional group	*p*
Number of the grafts				
LIMA/SV	101	34 (11/23)	67 (23/44)	
Patency rate at 1-week after surgery (%)				
LIMA		11/11 (100)	23/23(100)	–
SV		23/23 (100)	43/44 (97.7)	0.74
Patency rate at 1-month after surgery (%)				
LIMA		11/11 (100)	22/23 (97.7)	0.70
SV		22/23 (95.7)	42/44 (95.5)	0.56
Patency rate at 3-month after surgery (%)				
LIMA		11/11 (100)	22/23 (95.7)	0.70
SV		22/23 (95.7)	41/44 (93.2)	0.89
Stenosis degree at 1-week after surgery (%)				
LIMA		8.2 ± 4.3	8.7 ± 6.2	0.81
SV		9.2 ± 5.5	9.3 ± 6.0	0.96
Stenosis degree at 1-month after surgery (%)				
LIMA		11.5 ± 6.3	12.1 ± 5.8	0.78
SV		18.1 ± 6.3	19.2 ± 6.8	0.65
Stenosis degree at 3-month after surgery (%)				
LIMA		16.5 ± 6.7	16.8 ± 7.1	0.90
SV		22.3 ± 6.2	29.1 ± 7.3	0.01

## Complications

One case of gingiva bleeding and one case of arrhythmia were observed in the t-PA group; one case of hematochezia and two cases of arrhythmias were observed in the conventional group (*p* > 0.05) (Table 4).

**Table 4:** Postoperative follow-up indexes

Parameters	t-PA group (*n* = 12)	Conventional group (*n* = 25)	*p*
Arrhythmia (*n*)	1	2	0.81
BP (MAP), mmHg	125 ± 21	118 ± 19	0.31
Triglyceride (mmol/L)	2.09 ± 0.35	2.30 ± 0.69	>0.05
Cholesterol (mmol/L)	4.26 ± 1.20	4.14 ± 0.98	0.75
PLT (×10^9^/L)	207.2 ± 65.4	199.3 ± 87.2	0.78
Hemorrhage (*n*)	1 (gingiva)	1 (hematochezia)	0.81

## Discussion

The aim of this study was to explore the feasibility and safety of using t-PA to prevent graft restenosis after CABG. Results showed that restenosis of SV grafts was lower in the t-PA group at 3 months but not at 1 week or 1 month. The patency of the grafts was similar between the two groups. These results suggest that early application of t-PA after CABG might effectively prevent early restenosis of SV grafts.

t-PA is currently considered one of the most effective drugs for preventing and treating thrombotic diseases and is commonly used in thrombolytic therapy for acute myocardial infarction [21, 22]. A recent study also showed that t-PA could be used in patients with unstable angina to avoid coronary angioplasty, without significant complications [23]. Another study revealed that t-PA treatment decreased the 7-day mortality by 15% after CABG [24]. Previous studies have shown that the expression of t-PA is significantly downregulated in great SV as compared with the internal thoracic artery [25]. In addition, animal studies have demonstrated that the expression of t-PA in venous grafts is lower than that in arterial grafts, and that the anti-stenosis mechanisms of t-PA are mainly exerted at the early stage after transplantation [26]. Local transfection of t-PA to blood vessels inhibited early thrombogenesis after revascularization [27, 28]. In the present study, the degree of stenosis in SV grafts was significantly lower in the t-PA group than in the conventional group, which is supported by the anti-stenosis effects of t-PA in thrombolytic therapy [21, 22].

The present study is not without limitations. The small sample size and evaluation of the restenosis of the grafts at the early and middle stages after CABG are the main limitations of this study. Further clinical randomized trials with larger samples are needed to further validate the efficacies and long-term effects of the treatments.

## Conclusion

Early application of t-PA after CABG was feasible and safe, and might help prevent early restenosis of SV grafts. Additional clinical randomized trials are necessary to address this issue.
